# Comparative evaluation of Nano-Hydroxyapatite preparation and Calcium Sucrose Phosphate on microhardness of deciduous teeth after iron drop exposure - An in-vitro study

**DOI:** 10.4317/jced.53677

**Published:** 2017-04-01

**Authors:** Nilesh Rathi, Rutika Baid, Sudhindra Baliga, Nilima Thosar

**Affiliations:** 1 BDS, MDS, Dept. of Pedodontics & Preventive Dentistry, Sharad Pawar Dental College, Wardha-442004, Maharashtra, India; 2 BDS, Post graduate student, Dept. of Pedodontics & Preventive Dentistry, Sharad Pawar Dental College, Wardha-442004, Maharashtra, India

## Abstract

**Background:**

To evaluate and compare the microhardness of deciduous teeth treated with nano-hydroxyapatite and calcium sucrose phosphate after iron drop exposure.

**Material and Methods:**

Twenty healthy anterior deciduous teeth were collected and stored in 0.9% saline solution at room temperature. All the teeth were immersed in artificial saliva in an incubator shaker at 37° for an hour and then subjected to Vickers microhardness test at 100g load for 5 seconds. The teeth were then immersed in iron drop for 5 minutes, twice daily, rinsed with distilled water and kept in artificial saliva. This procedure was repeated for 7 days and teeth were subjected to microhardness testing. Further, the teeth were divided in two groups, each group containing 10 teeth. In group I, nanohydroxyapatite preparation and in group II, calcium sucrose phosphate were applied for 10 minutes, twice daily for 7 days and subjected again to microhardness testing again.

**Results:**

Vickers microhardness analysis revealed that iron drop exposure to teeth caused significant decrease in microhardness (*p*<0.05). Application of nanohydroxyapatite preparation in Group I showed significantly increased enamel microhardness (206.90) than that after iron drop exposure. Similarly, application of calcium sucrose phosphate in Group II showed significantly increased enamel microhardness (200.89) than that after iron drop exposure. Statistical difference was seen between the two groups, with nanohydroxyapatite preparation showing increased microhardness than calcium sucrose phosphate.

**Conclusions:**

Nanohydroxyapatite preparation and calcium sucrose phosphate have remineralizing effect over teeth affected by acid challenge of iron drops, nanohydroxyapatite preparation showing better results than calcium sucrose phosphate.

** Key words:**Iron drops, Nanohydroxyapaptite, calcium sucrose phosphate, anticay.

## Introduction

Children are affected by different types of blood dyscrasias and nutritional disorders. These blood disorders include pernicious anemia, sickle cell anemia, haemophilia etc. Iron deficiency anemia has been known to commonly affect the children causing high morbidity and mortality worldwide (1). Hence, paediatricians usually prescribe iron drops to infants and toddlers for the treatment. The citrate content in iron drop is highly acidic (2) and can cause erosion of teeth which decreases the enamel strength and accelerates the process of caries development in infants and toddlers (3). Deciduous teeth are more commonly affected because of less enamel thickness and mineralization. This altered structural arrangement results into lower microhardness of enamel in primary teeth as compared to permanent teeth (4,5). Hence, the use of various remineralizing agents is mandatory to prevent demineralization of teeth in such children.

Nano-hydroxyapatite has been advocated for remineralization of teeth. It is hydrophilic and has greater surface area than the conventional hydroxyapatite crystals. Hence, they have better wettability and form a thin but strong layer on enamel surface that bonds to tooth structure (6). Another remineralizing agent which is widely used is calcium sucrose phosphate which quickly breaks down into calcium, phosphate and sucrose ions. The common ion effect increases the rate of remineralization and repair on the superficial as well as the deep surfaces of enamel (7). So, the purpose of this study was to comparatively evaluate the effect of nano-hydroxyapatite preparation and calcium sucrose phosphate on microhardness of deciduous teeth after iron drop exposure.

## Material and Methods

Twenty non-carious anterior deciduous teeth were collected and stored in 0.9% saline.

-Nanohydroxyapatite preparation:

Nanohydroxyapatite powder was prepared in Central research laboratory, Jawaharlal Nehru Medical College, Sawangi (Meghe), Wardha, Maharashtra using in situ hybridization method. 4gm chitosan (HiMedia Laboratories Pvt. Ltd., Mumbai) was dissolved in 100 cc water with 2% acetic acid and mixed with an electric mixer for 24 hours. A purified 2M calcium nitrate tetrahydrate (S.D. Fine chem. Ltd., Thane) of 100 cc was added to it. Polyethylene glycol was added to control the growth of particles and this mixture was kept for high power mixing for 4 hours. Then, 100 cc of 2M diammonium hydrogen phosphate solution (Merck Specialities Pvt. Ltd., Mumbai) and 1g cetyltrimethyl bromide (HiMedia Laboratories Pvt. Ltd., Mumbai) were added to the solution which was titrated using sodium hydroxide to maintain pH at 11-12. The final solution was mixed for 24 hours to yield a homogenous suspension and filtered by centrifugation method at 3000 rpm. It was freeze-dried at -50°C to get a white nanohydroxyapatite powder.

-Microhardness testing:

A window of size 4x4 mm was marked on the buccal surface of teeth and the surrounding area was applied with varnish. The teeth were then mounted in acrylic with their prepared buccal surface 2 mm above the block. The window was polished to achieve a flat plane. Iron drop was diluted by adding artificial saliva in the ratio of 2:3 and the teeth were immersed in it as stated by Tabari et al. (8). Nano-hydroxyapatite powder and the silicone oil (Merck Specialities Pvt. Ltd., Mumbai) were mixed in a powder to liquid ratio of 1:3, so that it could be applied to the tooth structure. Calcium sucrose phosphate (Enafix, Group Pharmaceuticals, Ltd., Bangalore) was in paste form available commercially.

All 20 teeth were immersed in artificial saliva in incubator shaker at 37ºC for an hour. Then, the microhardness was tested using Vickers microhardness testing machine with a load of 100 g for 5 seconds at a displacement rate of 0.1µm, at BDCOE, Sevagram, Wardha. Microhardness was calculated for each sample based on the distance of 3 indentations on the smooth surface on each tooth and the mean of 3 values were calculated. All the teeth were immersed in iron drop (Tonoferon Drops, East India Pharmaceuticals Ltd., Kolkata) for 5 minutes, twice daily for 7 days and stored in artificial saliva. All the samples were subjected again to microhardness analysis. The teeth were then divided into two groups, Group I for application of nano-hydroxyapatite preparation and Group II for application of calcium sucrose phosphate, containing 10 samples each and application time was 10 minutes, twice daily for 7 days. The teeth were then subjected to microhardness analysis again.

-Statistical analysis:

Statistical analysis was done by using descriptive and inferential statistics using Students’ unpaired t test, one way ANOVA and Multiple Comparison Tukey Test. Analysis was done using SPSS software 17.0 version and *p*-value less than 0.05 was considered as level of significance.

## Results

**Figure 1:**
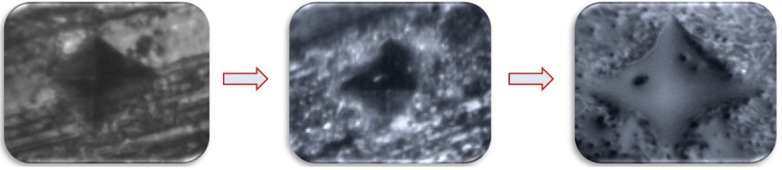
Pictures show indentations on (a) tooth enamel, (b) after iron drop exposure and (c) after application of nano-hydroxyapatite preparation (Group I) as seen in Vickers microhardness testing machine.

**Figure 2:**
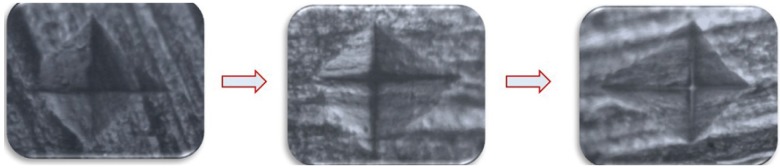
Pictures show indentations on (a) tooth enamel, (b) after iron drop exposure and (c) after application of calcium sucrose phosphate (Group II) as seen in Vickers microhardness testing machine.

**Figure 3:**
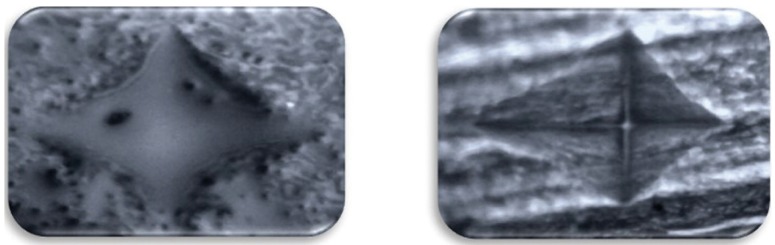
Pictures show indentations on tooth enamel after application of nano-hydroxyapatite preparation and calcium sucrose phosphate (Group I and II) as seen in Vickers microhardness testing machine.

In both the groups, teeth were subjected to microhardness analysis, then exposed to iron drops for 7days and subjected to micro-hardness analysis again. Mean microhardness of teeth was decreased after iron drop exposure from 197.79 ± 0.98 to 188.82 ± 1.17, which was found to be significant ( Table 1). Group I showed increase in mean microhardness of enamel from 195.34 ± 0.95 to 206.90 ± 2.15 after application of nano-hydroxyapatite preparation, which was significant ( Table 2). Similarly, Group II sho-wed increase in mean microhardness of enamel from 188.82 ± 1.17 to 200.89 ± 1.05 after application of calcium sucrose phosphate, which was again significant ( Table 3). On comparing the mean microhardness in Group I (206.90±6.82) and Group II (200.89±3.34), microhardness was found to be significantly increased in Group I ( Table 4), (Figs. 1-3).

**Table 1:**
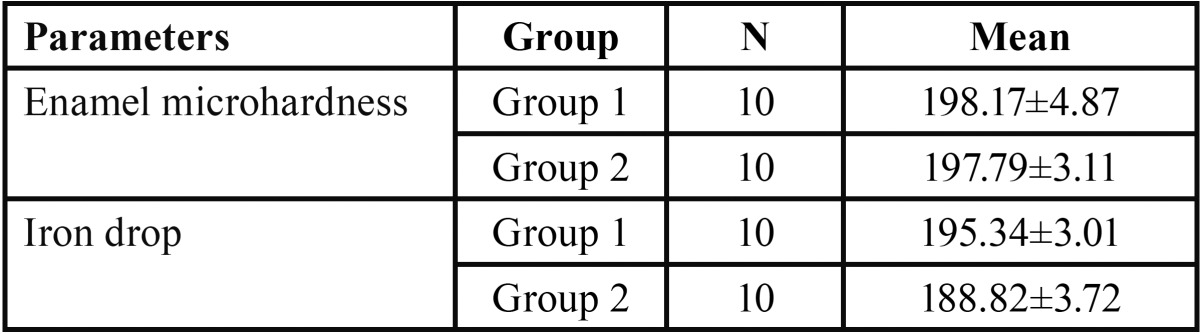
Mean microhardness of iron drops treated teeth samples in Group I and Group II by student’s unpaired ‘t’ test.

**Table 2:**
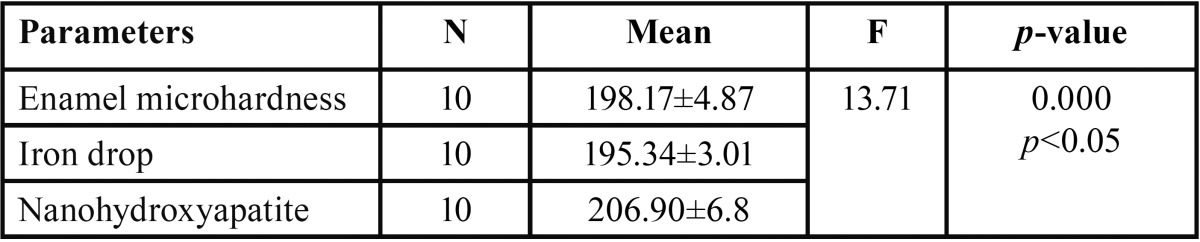
Comparison of mean microhardness of iron drops treated tooth enamel and after application of nano-hydroxyapatite preparation in Group I by one way ANOVA.

**Table 3:**
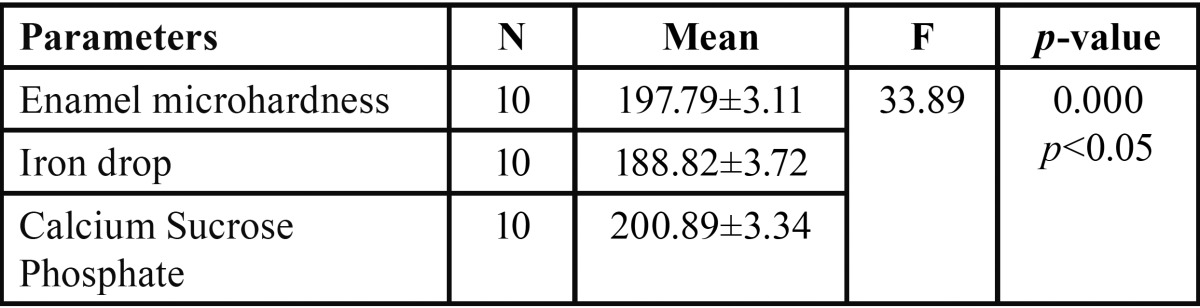
Comparison of mean microhardness of iron drops treated tooth enamel and after application of calcium sucrose phosphate in Group II by one way ANOVA.

**Table 4:**
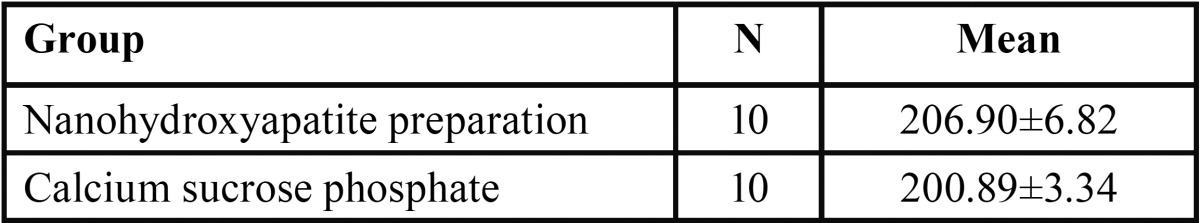
Comparison between mean microhardness of nano-hydroxyapatite preparation and calcium sucrose phosphate.

## Discussion

In present scenario, iron deficiency anemia has been found to be the most common nutrient deficiency in the world. It has caused impact on millions of lives, especially women and children, contributing to poor cognitive development, increased maternal mortality and decreased work capacity. Specifically, the intake of iron drops in children has shown clinically significant changes on their teeth surfaces. Low et al. (9) in his study on deciduous and permanent teeth found that the enamel of deciduous teeth is highly susceptible to demineralization, because of its higher organic content and low microhardness as compared to enamel of permanent tooth. Hence, the present study was conducted to assess the changes in enamel microhardness of deciduous teeth on application of iron drops and to compare the efficacy of remineralizing agents, nano-hydroxyapatite preparation and calcium sucrose phosphate.

Vickers microhardness test has been used in our study for assessing the microhardness of teeth on application of various remineralizing agents. This test gives accurate readings in both soft and hard materials i.e it gives identical hardness numbers on similar materials at different loads, while other tests need arbitrary changing of scales (10). The decalcified teeth lose the inorganic components leading to decreased hardness, but the accurate readings of this test made it our choice of analysis for microhardness.

James and Parfitt, 1953 (2) confirmed the erosive effects of iron drops that lead to decalcification and progression of caries into the enamel. Thakib *et al.* (11) found that different iron supplement products play cariostatic effect in the development of the dental caries. When the tooth is treated with iron salts, the precipitation of ferric phosphate occurs on the enamel surface leading to formation of an acid resistant coating of hydrous iron oxide. Alves *et al.* (12) found that the formation of hydrous iron coating has been proved to be highly erosive to tooth surface leading to low surface microhardness values. In our study also, exposure to iron drop decreased the microhardness of the enamel of the deciduous teeth rendering the need to introduce various remineralizing agents for repair of the tooth enamel.

Calcium sucrose phosphate, a commercially available remineralizing agent, has an agent anticay in its component, which is a mixture of calcium salts of sucrose phosphate esters and inorganic calcium orthophosphate (7). It breaks down into calcium, phosphate and sucrose phosphate ions and acts as a carrier for calcium and phosphate ions in water and saliva. Calcium and phosphate ions are rapidly adsorbed on enamel surface. These ions are mainly responsible for remineralization on tooth enamel by their common ion effect. Sucrose phosphate ion decreases rate of acid dissolution of hydroxyapatite and inhibits demineralization of tooth enamel. Sargod *et al.* (7) in their study, used Enafix toothpaste (containing calcium sucrose phosphate and agent anticay) on teeth affected by acid challenge due to use of demineralizing agents and found that its periodic application reduced the depth of enamel lesion on these teeth. Similar results were found in our study. It was seen that the enamel microhardness increased, in an iron drop treated tooth after application of calcium sucrose phosphate showing its remineralization effect.

Nanohydroxyapatite particles when compared to conventional hydroxyapatite particles are more hydrophilic in nature and have greater surface area. These properties of nanohydroxyapatite give them high wettability and help to form a thin but strong layer on enamel surface that binds to tooth structure (6). pH also plays an important role in effectiveness of nano-hydroxyapatite on tooth enamel. Acidic environment increases the solubility of nano-hydroxyapatite particles, making the solution saturated, which helps in better deposition on demineralised areas. At this pH, electrostatic force between the nano-hydroxyapatite particles and the tooth enamel is said to decrease, thus rendering better deposition. Release of higher concentrations of calcium and phosphate occurs, causing remineralization of the demineralized areas as proven in a study conducted by Tabari *et al.* (8).

Carious lesions in the enamel are more porous than the sound enamel, which renders better penetration of nanohydroxyapatite particles. These nanohydroxyapatite particles act as a template in precipitation process and attract large amounts of calcium and phosphate ions from the remineralizing solution, promoting calcium integrity and growth. Huang (13) in his study proved this remineralizing effect of nanohydroxyapatite particles, which proves fruitful in long term inhibition of caries process.

Enamel dissolution initiates at the prism-prism interface, under acidic conditions leading to caries formation. Li *et al.* (14) carried out a study using confocal laser scanning microscopy (CSLM) and found out that nanohydroxyapatite blocked Rhodamine B dye from entering the prism-prism sheath, proving that no enamel dissolution occurs after nanohydroxyapatite particles adhered to the tooth surface.

Calcium and phosphate ions in calcium sucrose phosphate aid in remineralization by forming hydroxyapatite particles [Ca10(PO4)6(OH)2, HAP]. Thus, hydroxyapatite and nano-hydroxyapatite particles have been used in various studies for reminera-lization of tooth and proved to be effective in various cases. Nano-hydroxyapatite particles have greater potential to remineralize when compared to conventional hydroxyapatite crystals (14) and are available as an ingredient in various mouthrinses, toothpastes and chewing gums. Huang in 2009 (13) and Swarup in 2012 (15) used fluoride in combination with nanohydroxyapatite and concluded that nanohydroxyapatite produced a new surface layer over the demineralised enamel, with morphology similar to that of biologic enamel. They also stated that 10% nanohydroxyapatite is optimal for remineralization of early carious lesions (13,15). Tabari M *et al.* (8) prepared nano-hydroxyapatite preparation by in-situ hybridization method and found that application of nano-hydroxyapatite preparation increased microhardness of tooth whether applied before or after iron drop exposure, but results were found better after the application of iron drop exposure. Application of nanohydroxyapatite preparation in our study also showed increase in microhardness of enamel after iron drop exposure.

In the present study, both remineralizing agents, nanohydroxyapatite preparation and calcium sucrose phosphate showed remineralizing effects on teeth treated with iron drops. But rendering to the size of hydroxyapatite particles, nanohydroxyapatite proved to be better in remineralization than calcium sucrose phosphate.

## Conclusions

Within limitations of the study, applications of both, nanohydroxyapatite plus silicone oil preparation and calcium sucrose phosphate, render remineralization of the teeth possible in the iron deficiency anaemia patients treated with iron drops. On comparison, nano-hydroxyapatite showed much better remineralizing effect than calcium sucrose phosphate.
